# Brain Acetaldehyde Exposure Impacts upon Neonatal Respiratory Plasticity and Ethanol-Related Learning in Rodents

**DOI:** 10.3389/fnbeh.2017.00039

**Published:** 2017-03-21

**Authors:** María B. Acevedo, Génesis D'Aloisio, Olga B. Haymal, Juan C. Molina

**Affiliations:** ^1^Laboratorio de Alcohol, Ontogenia y Aprendizaje, Instituto de Investigación Médica Mercedes y Martín Ferreyra, Consejo Nacional de Investigaciones Científicas y Técnicas (INIMEC-CONICET), Universidad Nacional de CórdobaCórdoba, Argentina; ^2^Experimental Psychobiology Chair, Department of Psychology, Universidad Nacional de CórdobaCórdoba, Argentina

**Keywords:** neonates, ethanol, acetaldehyde, breathing, operant learning

## Abstract

Prior studies indicate that neonates are very sensitive to ethanol's positive reinforcing effects and to its depressant effects upon breathing. Acetaldehyde (ACD) appears to play a major role in terms of modulating early reinforcing effects of the drug. Yet, there is no pre-existing literature relative to the incidence of this metabolite upon respiratory plasticity. The present study analyzed physiological and behavioral effects of early central administrations of ethanol, acetaldehyde or vehicle. Respiration rates (breaths/min) were registered at post-natal days (PDs) 2 and 4 (post-administration time: 5, 60, or 120 min). At PD5, all pups were placed in a context (plethysmograph) where they had previously experienced the effects of central administrations and breathing patterns were recorded. Following this test, pups were evaluated using and operant conditioning procedure where ethanol or saccharin served as positive reinforcers. Body temperatures were also registered prior to drug administrations as well as at the beginning and the end of each specific evaluation. Across days, breathing responses were high at the beginning of the evaluation session and progressively declined as a function of the passage of time. At PDs 2 and 4, shortly after central administration (5 min), ACD exerted a significant depression upon respiration frequencies. At PD5, non-intoxicated pups with a prior history of ACD central administrations, exhibited a marked increase in respiratory frequencies; a result that probably indicates a conditioned compensatory response. When operant testing procedures were conducted, prior ethanol or ACD central administrations were found to reduce the reinforcing effects of ethanol. This was not the case when saccharin was employed as a reinforcer. As a whole, the results indicate a significant role of central ACD upon respiratory plasticity of the neonate and upon ethanol's reinforcing effects; phenomena that affect the physiological integrity of the immature organism and its subsequent affinity for ethanol operationalized through self-administration procedures.

## Introduction

Low doses of ethanol (0.11 g/kg/h), combined or not with a tocolytic agent (ritodrine), have been employed to reduce the incidence of human preterm births. Under these clinical conditions, in approximately 80% of the patients, uterine contractions were suppressed and a significant number of preterm births were prevented (Schrock et al., 1989). According to this study no adverse effects of ethanol were observed. Yet, the obstetric use of ethanol, depending upon factors such as dose, frequency of exposure and fetal stage of development has been questioned due to a variety of disruptive physiological effects of the drug upon the fetus. Hypothermia, acidosis, hypercapnia, bradycardia, hypoglycemia, apneas and hypoxia are likely to occur in the developing organism exposed to ethanol (Abel, 1981; Duxbury, 2001; Abate et al., 2004). When considering the central nervous system, there are also numerous studies confirming disruptions caused by relatively infrequent and small doses of the drug upon a variety of parameters. Rat fetuses exposed to low ethanol doses (blood ethanol concentration ≤ 30 mg%) show impairments in spatial learning accompanied by alterations in hippocampal glutamate-dependent synaptic neurotransmission (Savage et al., 2002). In rhesus monkeys, moderate maternal ethanol consumption (0.6 g/kg ethanol daily) during midgestation to late gestation, induces heightened dopaminergic function (Wise, 2002). During a stage in the development of the mouse characterized by a brain growth spurt, similar to the one observed during the third gestational trimester in humans (Dobbing and Sands, 1973, 1979), a single ethanol dose (0.63 g/kg) yielding relatively low peak blood ethanol levels (57 mg%) is sufficient to trigger a significant neuroapoptosis response (Young and Olney, 2006).

Preclinical and clinical studies have indicated that relatively low ethanol doses during pregnancy are sufficient to trigger fetal sensory and learning capabilities with an impact upon later patterns of chemosensory recognition of the drug, ethanol odor and taste preference (Faas et al., 2000, 2015; Abate et al., 2008) and sensitivity to the drug's positive reinforcing effects (Nizhnikov et al., 2006). Fetal experience with ethanol generates conditioned responses derived from the association between the drug's odor and taste and its motivational properties (Abate et al., 2001; Spear and Molina, 2005; Molina et al., 2007; Cullere et al., 2015). These phenomena predispose the organism to heightened seeking and intake patterns of the drug during infancy and adolescence (Dominguez et al., 1998; Foltran et al., 2011; Fabio et al., 2013; Acevedo et al., 2017). Epidemiological studies have validated the significant association existing between fetal ethanol exposure and subsequent predisposition to seek and consume the drug (Baer et al., 1998, 2003; Griesler and Kandel, 1998; Yates et al., 1998; Alati et al., 2006).

Acetaldehyde (ACD), ethanol's principal metabolite, mainly and rapidly forms in the perinatal brain via the oxidative process of the catalase system. The activity of this enzymatic system is significantly higher during early ontogeny relative to adolescence and adulthood (Del Maestro and McDonald, 1987; Gill et al., 1992; Hamby-Mason et al., 1997). When considering ethanol's reinforcing effects, ACD formation in the brain plays a critical role (Wall et al., 1992; Hahn et al., 2006). In newborn rats, intracisternal administration of relatively low doses of ethanol (100 mg%) or of ACD (0.35 μmol) promote appetitive conditioning (Nizhnikov et al., 2007; March et al., 2013a,b). Furthermore, when considering either peripheral or central administration of ethanol, the establishment of early appetitive memories are blocked when sequestering brain ACD via the use of d-penicilamine (Pautassi et al., 2011; March et al., 2013a,b) or when inhibiting the catalase system through sodium azide (Nizhnikov et al., 2007).

Ethanol consumption during pregnancy has also been found to endanger the wellbeing of the fetus and the neonate due to its detrimental effects upon the respiratory system and its plasticity; a phenomenon that has stimulated research efforts based on the fact that fetal alcohol exposure is a risk factor for Sudden Infant Death Syndrome (Burd et al., 2004; O'Leary et al., 2013). In human and ewes, the depressant effects of the drug upon fetal breathing movements (FBMs) have been well documented (Vojcek et al., 1988; Brien and Smith, 1991) and there is evidence that maternal human consumption of only two glasses of wine during late gestation significantly suppresses fetal breathing activity (Brien and Smith, 1991; Dillner et al., 1996). In rats, chronic ethanol exposure during pregnancy, reduces brainstem-dependent respiratory rhythmic activity in the progeny and sensitizes juveniles to the depressant effects of acute ethanol upon phrenic and hypoglossal nerve activity (Dubois et al., 2006). Analogous effects in rats have been recently reported utilizing moderate levels of ethanol exposure during the last days of pregnancy or during the first days of post-natal life (Cullere et al., 2015; Macchione et al., 2016; Acevedo et al., 2017). These stages in development, in terms of brain developmental patterns, are equivalent to the 2nd and 3rd human gestational trimester; respectively (Dobbing and Sands, 1973, 1979). Indeed, we have reported that maternal intragastric (i.g.) administration of ethanol (2.0 g/kg) during gestational days (GDs) 17–20 is sufficient to sensitize the progeny to the drug's depressant effects upon respiratory rates and exacerbate the presence of apneic episodes; disruptions that occur without affecting different pulmonary morphometric parameters (Cullere et al., 2015). This sensitization process has also been observed when neonates [post-natal days (PDs) 3, 5, and 7] were peripherically administered with ethanol (i.g.: 2.0 g/kg). Furthermore, in both studies, it was observed that the explicit association between ethanol's sensory attributes and the depressant consequences of the drug resulted in conditioned isodirectional breathing responses (Macchione et al., 2016).

After systematically reviewing the pre-existing literature concerning ethanol's central effects upon breathing patterns of the perinate, we were unable to find specific literature related with possible contributions of ACD. Despite this observation, it should be noted that respiratory plasticity is linked with thermoregulatory disruptions. Indeed, prenatal or neonatal hypothermia can cause respiratory arrests (Duxbury, 2001). Among the multiple physiological consequences of ACD is the modulation of the thermoregulatory system. In mice, peripheral administration of ACD causes hypothermia (Closon et al., 2009). Nevertheless, there is certain degree of contradiction relative to the prior statement. It has been observed that in rats the inhibition of the central catalase system following ethanol administration seems not to play a significant role in the induction of hypothermia (Aragon et al., 1991).

The first goal of the present study was based on the preceding observations: (i) the absence of specific literature related with the central role of ethanol or its first metabolite (ACD) in terms of disruptive effects upon early respiratory plasticity and (ii) a possible association existing between ethanol and/or ACD central effects leading to thermoregulatory alterations that may impact upon breathing responsiveness. To address these phenomena in perinatal rats, it was decided to employ similar central ethanol (100 mg%) and ACD (0.35 μmol) doses that have been observed to exert analogous motivational effects (Nizhnikov et al., 2007; March et al., 2013a,b). A second goal was to further analyze if central pre-exposure to the drug or its metabolite modulate subsequent seeking behavior of ethanol as a reinforcer in an operant task specifically developed for perinatal or infant rats (Arias et al., 2007; Bordner et al., 2008; March et al., 2009; Miranda-Morales et al., 2014). These goals were sequentially examined. As a first step, during PDs 2 and 4, pups were intracisternally administered with either ethanol, ACD or a phosphate buffer as a control solution and respiration rates were recorded at different post-administration times. At PD5, all pups were re-exposed to the respiratory testing chamber without receiving any specific drug. This particular strategy obeys to the fact that prior experiments have indicated that early respiratory plasticity is also dependent upon exteroceptive ambient cues originally associated with breathing pattern changes. Following this testing procedure, pups were evaluated in terms of operant responding regulated by either an ethanol solution or a sweet reinforcer (saccharin). The inclusion of this last reinforcer obeyed to the need to control for unspecific learning alterations derived from the preceding central drug administration experiences. Body temperatures, before and after each specific drug treatment or evaluation procedure, were recorded.

## Materials and methods

### Subjects

A total of 146 Wistar neonate rats, representative of 17 litters, were employed. Rats were born and reared at the vivarium of the Instituto de Investigación Médica Mercedes y Martin Ferreyra (INIMEC-CONICET-UNC, Argentina). The colony room was illuminated on a 12 h light/dark cycle (lights on: 08:00–20:00) at an ambient temperature and humidity of 22 ± 1°C and 45%, respectively. Births were daily examined and the day of parturition was considered post-natal day 0 (PD0). At PD1 each litter was randomly culled to 10 pups (5 males and 5 females, whenever possible). Throughout days pups were kept with their dams in standard cages that contained water and food *ad libitum* (ACA Nutrición, Buenos Aires, Argentina).

All experimental treatments were in accordance with the Guide for Care and Use of Laboratory Animals (National Research Council, 1996) and were approved by the Institutional Animal Care and Use Committee of our institution (CICUAL-INIMEC-CONICET-UNC). To reduce confounds between litter and treatment effects (Holson and Pearce, 1992) no more than one male and one female per litter were assigned to a given experimental condition.

### General experimental procedures

During PDs 2 and 4, pups were removed from their maternal cages and placed in similar cages partially filled with clean corncob. Ambient temperature was kept at 31–33°C via heating pads placed beneath the cages. Pups were centrally administered with a buffer solution, ethanol or ACD (see below) and tested in a plethysmograph at post-administration time 5, 60, or 120 min. Respiratory frequencies were assessed during 5 consecutive minutes. At PD5 pups representative of each prior treatment were removed from their maternal cages and kept in pairs under the same holding conditions as in the previous experimental days. Fifteen minutes later, respiratory evaluations were performed. Following these physiological recordings, pups were subjected to a minor surgical procedure in order to implant an intraoral cannula that served to conduct operant conditioning procedures defined by saccharin or ethanol reinforcement. Body temperatures were recorded before and after each specific physiological or behavioral evaluation.

### Central drug administration procedures

Ethanol (100 mg%), acetaldehyde (0.35 μmol) and phosphate buffer (PB 0.1M) were administered with a 30 gauge hypodermic needle attached to a 20-cm length of polyethylene-10 tubing (PE-10 Clay Adams, Parsippany, New Yersey, USA) connected to a 50 μl gastight syringe (Hamilton, Reno Nevada, USA). Fluids (1 μl) were slowly injected (5–8 s) into the foramen magnum between the occipital bone and the first cervical vertebra, with the needle tip placed 1.5 mm depth in the cisterna magna (IC). PB 0.1M served as vehicle for ethanol and acetaldehyde solutions. The needle was kept in position for 10 s. The appearance of a small quantity of cerebrospinal fluid served to indicate the successful placement of the administrations. Similar procedures have been previously utilized in different studies (Varlinskaya et al., 1996; Petrov et al., 1998; Nizhnikov et al., 2006, 2007; March et al., 2013a,b).

### Determination of breathing frequencies

Breathing frequencies were determinated through a whole body plethysmograph (Model 10G equipped with the software “Breath Medidor de Respiración,” Itcom, Argentina). The apparatus was built to record breathing patterns of small organisms weighing between 6 and 28 g. It consists of two identical transparent and hermetic Plexiglas chambers (5 × 10 × 5 cm), that are interconnected via a polyurethane hose system. The hose system allows injection and extraction of equivalent amounts of air in both chambers in order to maintain constant and equivalent pressures. One of the chambers is used as a testing device while the other serves as a reference box in terms of flow/air pressure. The plethysmograph records air pressure/flow rate differences between the testing and reference chambers. These differences activate a pressure sensor (AWM2100 Honeywell) with the capability of recording one complete breathing event every 1 × 10^−7^ s. The plethysmograph records the breathing response every 1.5 s. These scores are transformed to mean breaths per minute.

For each session, unrestrained awake pups were introduced into the chambers and the lids were closed. One minute after that pups were individually placed inside the chamber, respiratory responses were measured during 10 consecutive minutes. The minute of delay at the beginning of the test was used to allow air pressure stabilization in the chamber.

An air conditioner kept the room temperature at 22 ± 1°C during experimental sessions. The temperature was kept at 31–33°C (similar to their maternal nest thermal condition) inside the plethysmograph chamber through heating pads placed beneath the apparatus (Julien et al., 2010). The overall procedure has been previously used to evaluate breathing disruptions as a function of pre- and post-natal ethanol exposure (Cullere et al., 2015; Macchione et al., 2016; Acevedo et al., 2017).

### Body temperature measurements

Body temperatures were non-invasively registered through a thermal infrared imaging camera (“Flir Exx Series,” Boston FLIR System, Inc.). The temperature corresponding to the nape of the neck of each subject served as the dependent variable. Thermal measurements were taken before and after each plethysmograph recording (PDs 2, 4, and 5) as well as prior and following each operant conditioning test (PD 5).

### Apparatus and operant conditioning test

At PD5, and following breathing evaluations, pups were removed from their maternal cages and were intraorally implanted with a cannula (PE-10) that allowed liquid infusions (Hall, 1979; Dominguez et al., 1996; Abate et al., 2001; Cheslock et al., 2001; Arias et al., 2007; Bordner et al., 2008; Miranda-Morales et al., 2014). They remained pair-housed in holding cages for 3 h until operant procedures took place. Before commencement of the evaluation, animals were anogenitally stimulated with a cotton swab to promote urination and defecation, weighed to the nearest 0.01 g. They were then fastened inside a disposable respirator mask (3M dust, fume and mist respirator 8801 P2) through a restrictor vest, expandable enough, to allow free movements of the head and limbs. The respiration mask was tilted at 40 degrees from the floor surface supported with a cardboard box (see Arias et al., 2007 for further procedural details).

All procedures took place at a constant temperature (31–33°C) via the use of heating pads. A 40–50-cm section of polyethylene-50 tubing (PE-50 Clay Adams, Parsippany, New Yersey, USA) was connected to the end of oral cannula (PE-10) and to a 5 ml syringe (Becton Dickinson, Rutherford, NJ) with a 23-gauge needle that was filled with a specific solution and mounted in an infusion pump (KD Scientific, Model 200, Holliston, MA). The pump was set to deliver 1 μl of fluid in 1 s directly into the intraoral cavity of a given “Paired” pup and its corresponding “Yoked” control (see description below). Once evaluations begun, pups were able to gain access to intraoral infusions of 0.1% w/v saccharin or 3% v/v ethanol solution (Porta Hnos, Córdoba, Argentina; vehicle: tap water).

To this end, two same-sex and same drug-treatment pups from a single litter with similar body weights were placed in front of a touch-sensitive copper sensor (5 cm length × 1 cm width × 45 cm 1 mm thickness). The sensor was 1 cm away from their mouths and perpendicular to the floor while they remained hold inside the mask. Each time the animal touched the sensor a red light bulb lit signaling a physical contact, which resulted in an infusion pump pulse.

The apparatus was set to work with two subjects at a time: a Paired animal receiving infusions in a fixed ratio (FR) 1 schedule and a Yoked control receiving infusions in accordance to its corresponding paired pup. Each evaluation lasted 15 min. During these sessions pups received a given solution (ethanol or saccharin reinforcers) contingent upon their operant behavior (i.e., sensor contact). All pups received 4 priming pulses at the beginning of the training session, 60, 120, and 180 s. These pulses were administered independently of motor activity patterns in order to introduce the pup with the reinforcer and to minimally stimulate head and body movements. The number of sensor contacts of each Paired subject and its corresponding Yoked control were recorded. Similar procedures have been employed when analyzing early operant leaning regulated by positive reinforcers such as milk, sucrose and ethanol (Arias et al., 2007; Bordner et al., 2008; March et al., 2009; Miranda-Morales et al., 2014).

### Experimental design and data analysis

Body weights were analyzed using a four-way mixed analysis of variance (ANOVA). Drug treatment at PDs 2 and 4 (PB 0.1 M, ACD 0.35 μmol or ethanol 100 mg%), sex (male or female) and post-administration time (5, 60, or 120 min) served as between-group factors. Days of assessment (PDs 2, 4, and 5) served as the within-measure factor. A five-way mixed ANOVA was performed to analyze mean respiration rates at PDs 2-4 where drug treatment, sex and post-administration time served as the independent factors, while days of assessment and minutes corresponding to each specific evaluations (minutes: 1–5) represented repeated measures. At PD5 breathing patterns were analyzed through a between-within ANOVA defined by the same independent factors and repeated measures.

Thermoregulatory processes were analyzed using a five- or four-way mixed ANOVA (PDs 2, 4, and 5; respectively) where drug treatment, sex, post-administration time served as between factors while post-natal days and time of temperature recordings (before and after plethysmograph assessments) were considered as within-group variables.

Relative to the operant task, the total number of sensor contacts was considered the dependent variable. Separate ANOVAs were conducted to analyze operant performance when either saccharin or ethanol served as reinforcers. More specifically, a two-way mixed ANOVA was utilized. This inferential analysis was defined by prior drug treatments the between-group factor and conditioning status (Paired or Yoked) as the within-group factor.

Preliminary analysis relative to operant performance indicated no significant main or interaction effects when considering sex as a factor. Therefore, data were collapsed across sex for all the remaining analyses. The absence of sex effects has also been observed in prior studies when employing a variety of reinforcers in operant conditioning tasks during early ontogeny (Bordner et al., 2008; March et al., 2009; Miranda-Morales et al., 2010, 2012a,b, 2014).

The loci of significant main effects were further analyzed with Ducan's *post-hoc* tests. A rejection criterion of *p* < 0.05 was adopted for all statistical analyses in the present study. According to the nature of the dependent variables under consideration, tests were performed using between or within error terms. Since there is no unambiguous choice of appropriate error term for *post-hoc* comparisons involving between- and within-group significant interactions (Winer, 1991), orthogonal planned comparisons were conducted when such interactions were obtained. All the statistical analyses were performed using the STATISTICA 8.0 software.

## Results

### Body weights across days (PDs 2, 4, and 5)

Data corresponding to body weights across days was analyzed via a between-within ANOVA (drug treatment at PDs 2 and 4 × post-administration time × post-natal days × sex). As expected body weights progressively increased as a function of age; *F*_(2, 250)_ = 3,897.01, *p* < 0.0001. Duncan's *post-hoc* tests showed that pups at PD4 exhibited significantly greater body weights than those previously recorded during PD2. At PD5, weight values were significantly higher than those observed at PD4 (means ± standard errors of the means for each day were as follows: PD2, 7.25 ± 0.06 g; PD4, 10.08 ± 0.07 g and PD5, 11.41 ± 0.09 g. Body weights did not differ as a function of the other factors under consideration.

### Breathing frequencies during drug pretreatment (PDs 2 and 4) and test (PD5)

**Figure 1:**
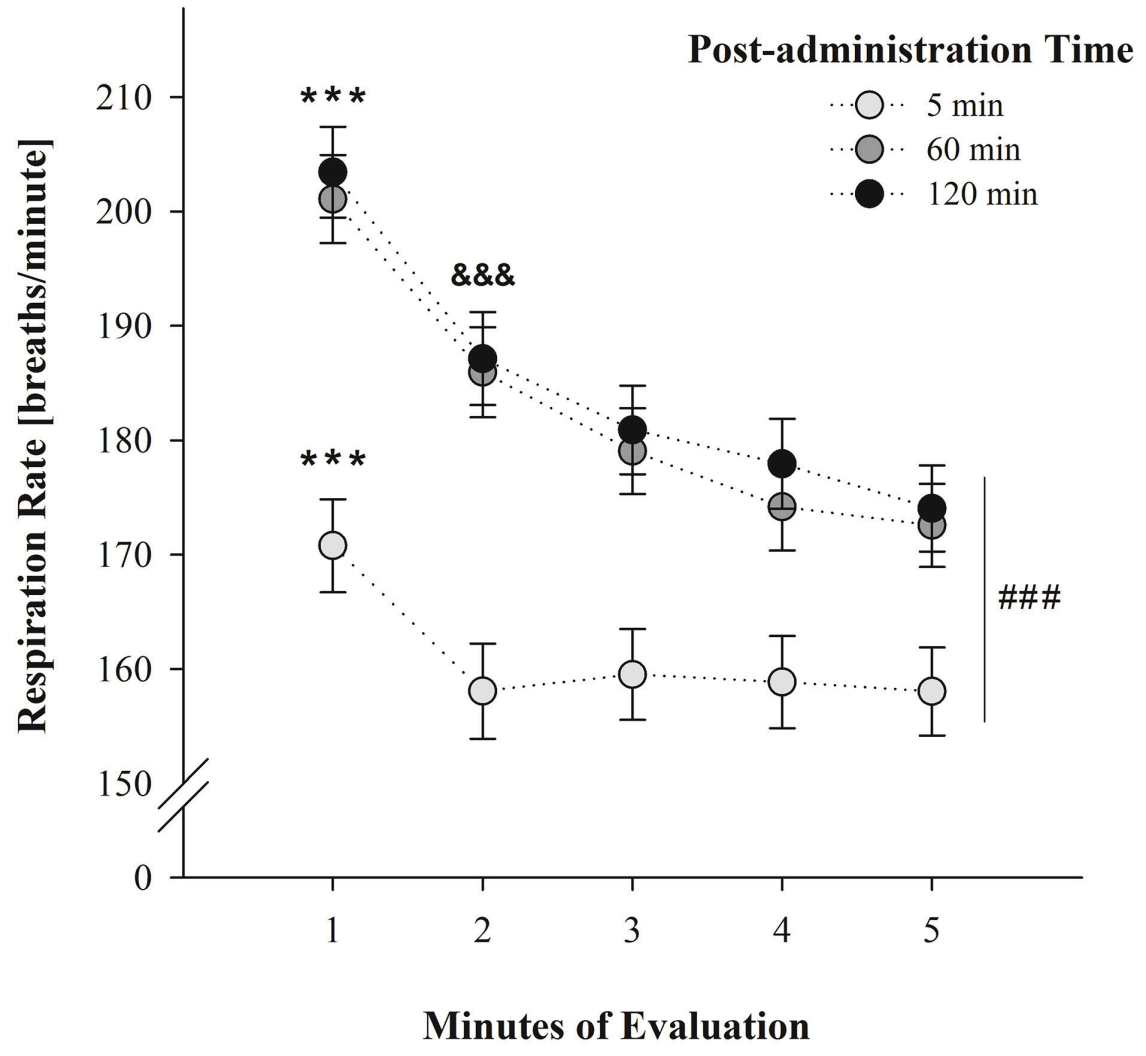
**Breathing rates (breaths/min) as a function of post-administration time (5, 60, or 120 min) and minutes of evaluation**. Data have been collapsed across sex, postnatal days and drug treatment. ^###^ Indicates significant differences between breathing scores at post-administration time 5 min relative to scores attained at post-administration time 60 and 120 min. ^***^ Indicates significant differences between respiratory rates at minute 1 relative to the remaining minutes of evaluation; *p* < 0.0001. ^&*&&*^ Indicates significant differences between minute 2 and minute 5; *p* < 0.0001. Vertical lines indicate standard errors of the means (SEMs).

Figure 1 illustrates average breathing responses corresponding to PDs 2 and 4 as a function of post-administration time (5, 60, or 120 min) and across minutes of evaluation. Respiration rates were not significantly different across days. The between-within ANOVA [drug treatment (PB 0.1 M, ACD 0.35 μmol or ethanol 100 mg%) × sex (female or male) × post-administration time (5, 60, or 120 min) × days of assessment (PDs 2 and 4) × minutes of evaluation (1–5)] indicated significant main effects of sex *F*_(1, 125)_ = 5.90, *p* = 0.0158; post-administration time *F*_(2, 125)_ = 12.27, *p* < 0.0001; minutes of evaluation *F*_(4, 500)_ = 118.49, *p* < 0.0001 as well as significant interactions between post-administration time and minutes of evaluation *F*_(8, 500)_ = 7.48, *p* < 0.0001. Drug treatment was also found to significantly interact with post-administration time and minutes of evaluation; *F*_(16, 500)_ = 2.13, *p* = 0.0063.

According to Duncan's *post-hoc* tests, breathing frequencies were significantly higher in male than female pups (181.30 ± 2.99 and 170.91 ± 3.01 breaths/min; respectively). Relative to the significant main effects of post-administration time, minutes of evaluation, and its significant interaction at PDs 2 and 4; *post-hoc* tests showed that breathing responses were significantly lower at post-administration time 5 min relative to the scores attained at 60 and 120 min. It is likely that the stress related with the intracisternal administration of the drugs, affected breathing rates shortly after performing these procedures. At 60 and 120 min, respiratory frequencies were similar to those reported in previous experiments where a given vehicle (e.g., water) was intragastrically administered 30 min prior to the evaluation (Macchione et al., 2016; Acevedo et al., 2017).

Within each test, respiration rates decreased as a function of the progression of the test; a phenomenon probably indicative of habituation to the context. This progressive depression was particularly observed in the group of animals evaluated at 60 and 120 min. When the evaluation was conducted 5 min after drug administration pups exhibited heightened respiratory rates during the initial minute of the test relative to the remaining minutes. This interaction has been depicted in Figure 1.

**Figure 2:**
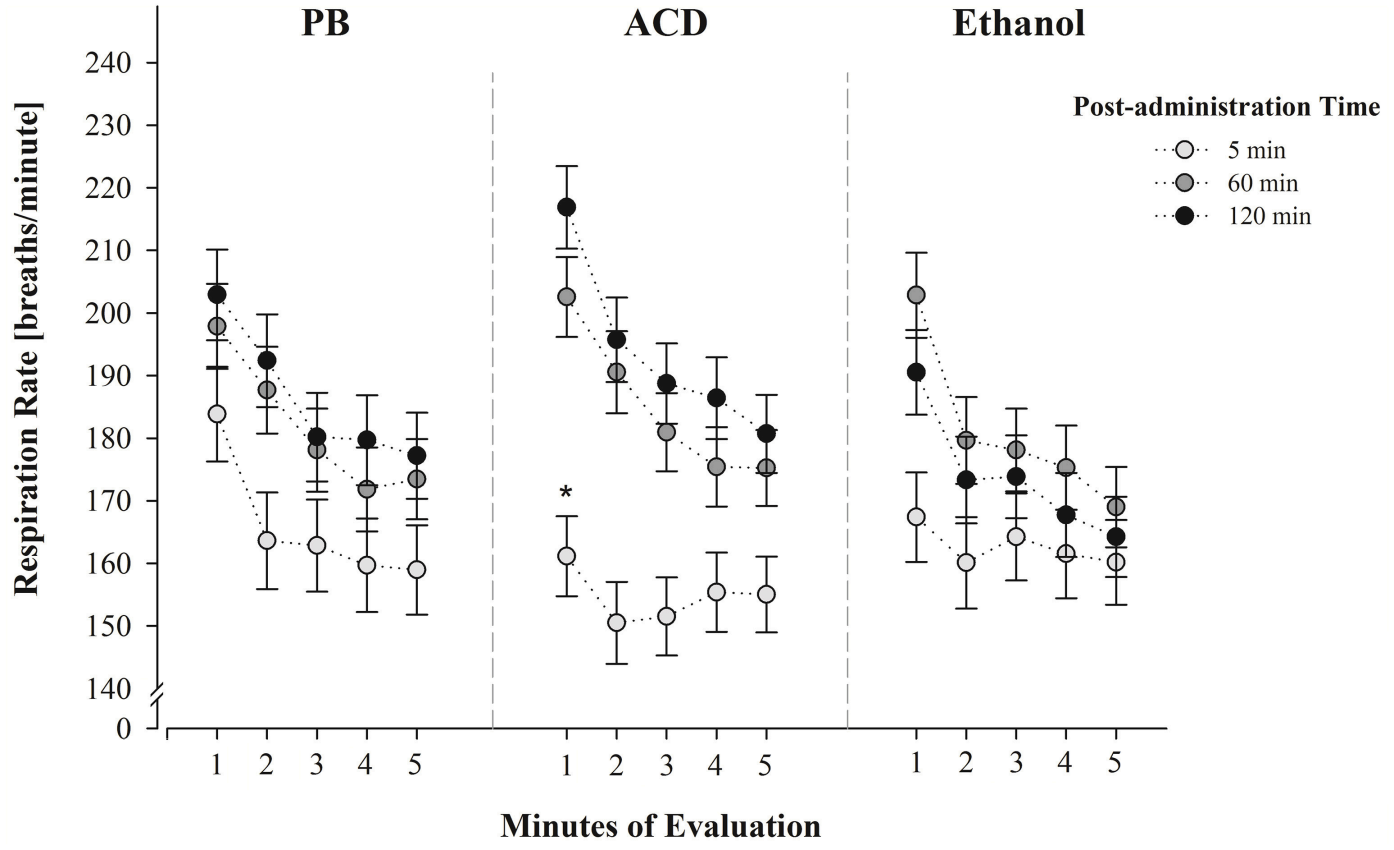
**Respiration frequencies (breaths/min) at PDs 2 and 4 as a function of drug treatment (PB, ACD or Ethanol), post-administration time (5, 60, or 120 min) and minutes of evaluation (1–5)**. Data have been collapsed across sex and postnatal days. ^*^ Indicates a significant difference between PB-treated and ACD-treated pups (post-administration time: 5 min.; minute of evaluation: 1 min); *p* < 0.05. Vertical lines indicate standard errors of the means (SEMs).

With regard to the triple interaction involving drug treatment, post-administration time and minutes of evaluation, planned comparisons indicated significant differences in respiration rates between PB-treated and ACD-treated pups during the first minute of evaluation (Figure 2). This effect was only observed 5 min after administering the drug. At this point in time, ethanol-treated animals showed intermediate respiratory frequencies relative to PB- and ACD-treated pups.

At PD 5, the corresponding between-within ANOVA (drug treatment at PDs 2 and 4 × sex × post-administration time × minutes of evaluation) showed that breaths per minute significantly varied as a function of drug treatment [*F*_(2, 128)_ = 4.37, *p* = 0.0146], minutes of evaluation [*F*_(4, 512)_ = 110.95, *p* < 0.0001] and the following two-way interaction: drug treatment × minutes of evaluation [*F*_(8, 512)_ = 1.99, *p* = 0.0447].

**Figure 3:**
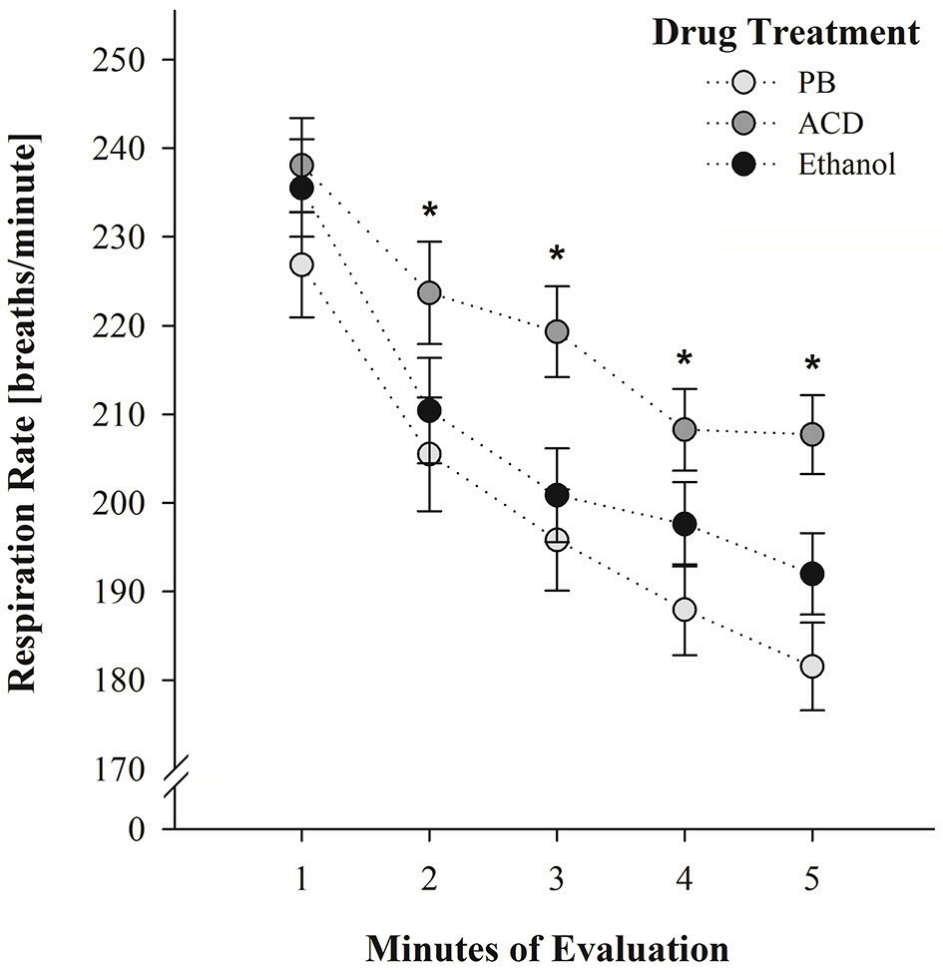
**Breathing rates (breaths/min) as a function of drug treatment (PB, ACD, or Ethanol) and minutes of evaluation at PD 5**. ^*^ Indicates significant differences between ACD pups and pups pre-exposed to PB administrations. Vertical lines indicate standard errors of the means (SEMs).

Once, again breathing responses progressively decreased as a function of the passage of time. Moreover, the group of animals previously treated with acetaldehyde during PDs 2 and 4 exhibited significantly higher breathing frequencies relative to the control group (PB). This significant difference was observed at minutes 2, 3, 4, and 5. Breathing scores of ethanol-treated pups did not significantly differ from PB- or ACD-treated subjects throughout the evaluation. This interaction has been depicted in Figure 3. As can be observed ACD-treated pups exhibited during PD5 breathing patterns which were opposite to those recorded at PDs 2 and 4 at the earliest post-administration time (5 min). When tested without being administered with acetaldehyde (PD5) breathing frequencies were significantly higher than in controls while under the effects of the drug (PDs 2 and 4), respiration rates were significantly lower.

### Thermal responsiveness during drug treatment (PDs 2 and 4) and test (PD 5)

As stated, body temperatures of pups treated with PB 0.1M, ACD 0.35 μmol or ethanol 100 mg% were recorded immediately before and after being exposed to the plethysmograph at PDs 2 and 4 as well as at PD5 (see Table 1). The corresponding between-within ANOVA (drug treatment × sex × post-administration time × days of assessment x moment of recording) during PDs 2 and 4 only indicated a significant main effect of post-administration time; *F*_(2, 117)_ = 92.26, *p* < 0.0001. Thermal temperatures soon after drug treatment (5 min) were significantly lower (33.91 ± 0.12°C) than those observed at post-administration times 60 and 120 min (35.96 ± 0.11 and 35.62 ± 0.12°C; respectively) Apparently, a stress-related factor derived form intracerebral administrations was responsible for the significant decrease in body temperature in pups tested 5 mins after the injection. At PD 5 (drug treatment × sex × post-administration time × moment of recording) no significant differences emerged when considering the main factors and the interactions between them. Notice that at this age pups were not IC administered. A similar lack of main significant effects or interactions was observed when processing body temperatures before and after the operant task.

**Table 1:** **Pup's body temperatures across days as a function of drug treatment and post-administration time**.

**Drug treatment at PDs 2 and 4**	**Post-administration Time (min)**	**Body temperature (°C)**					
		**PD2**		**PD4**		**PD5**	
		**Before**	**After**	**Before**	**After**	**Before**	**After**
Phosphate buffer (PB 0.1M)	5	34.23 ± 0.31	34.12 ± 0.29	33.96 ± 0.27	34.05 ± 0.28	34.99 ± 0.27	34.77 ± 0.28
	60	35.73 ± 0.30	36.01 ± 0.28	35.81 ± 0.26	35.66 ± 0.27	34.91 ± 0.24	35.06 ± 0.25
	120	35.67 ± 0.31	35.86 ± 0.29	35.56 ± 0.27	35.40 ± 0.28	34.66 ± 0.26	34.58 ± 0.27
Acetaldehyde (0.35 μmol)	5	33.83 ± 0.28	34.12 ± 0.26	33.71 ± 0.25	33.83 ± 0.25	34.72 ± 0.24	34.57 ± 0.24
	60	36.23 ± 0.27	36.35 ± 0.25	35.98 ± 0.23	35.83 ± 0.24	35.12 ± 0.23	34.38 ± 0.24
	120	35.68 ± 0.29	35.36 ± 0.27	36.04 ± 0.26	35.49 ± 0.26	34.64 ± 0.23	34.96 ± 0.24
Ethanol (100 mg%)	5	33.83 ± 0.30	34.12 ± 0.28	33.42 ± 0.27	33.63 ± 0.28	34.43 ± 0.26	34.17 ± 0.26
	60	36.21 ± 0.26	36.07 ± 0.25	35.70 ± 0.23	35.87 ± 0.24	35.18 ± 0.23	34.65 ± 0.24
	120	35.76 ± 0.30	35.53 ± 0.28	35.78 ± 0.26	35.28 ± 0.27	34.57 ± 0.25	34.39 ± 0.25

*Values are expressed as means ± SEMs. Body temperatures at PDs 2 and 4, recorded immediately after intracisternal administrations (5 min), were significantly lower than those registered at 60 or 120 min*.

### Operant conditioning at PD5

**Figure 4:**
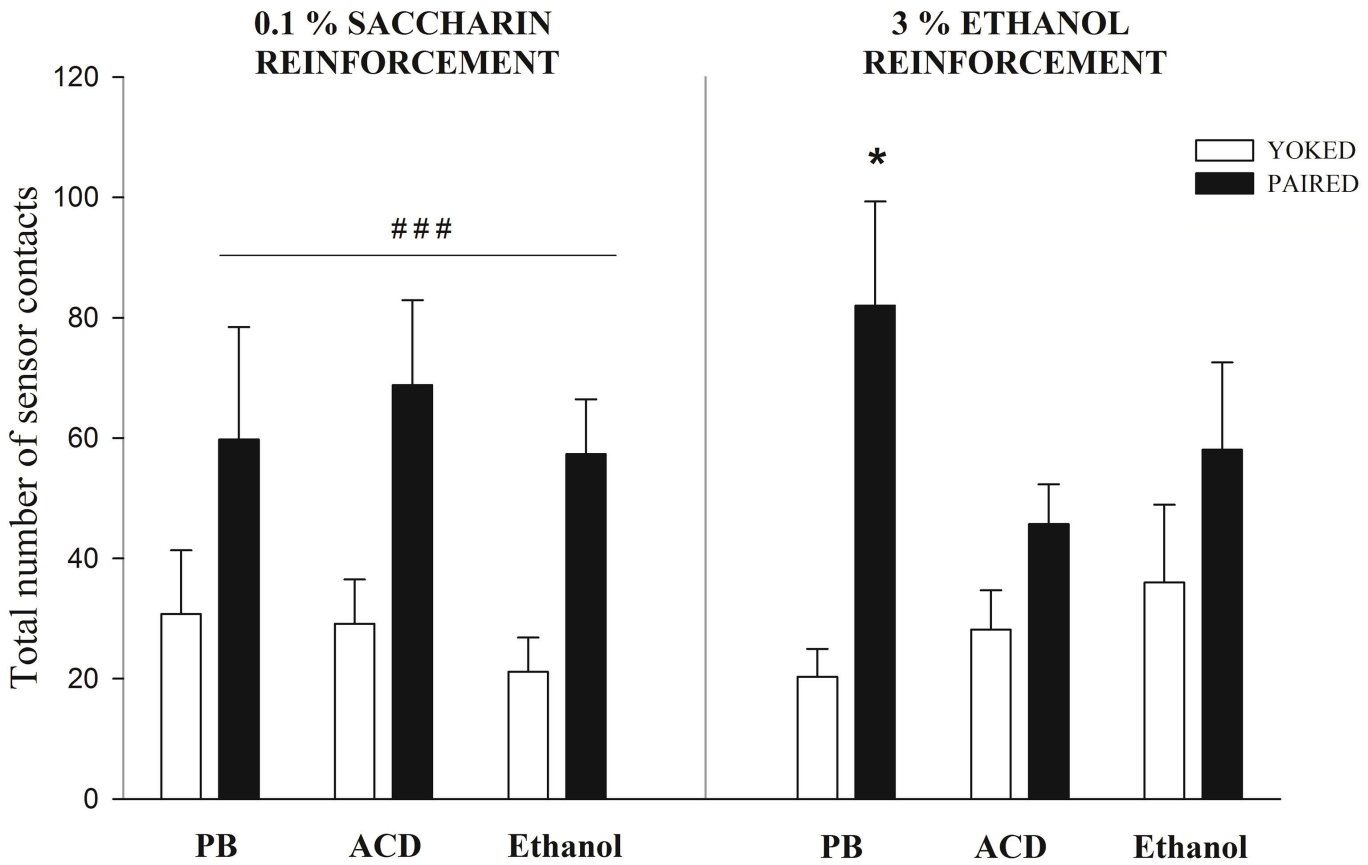
**Sensor contacts at PD5 when pups were reinforced with saccharin or ethanol**. When saccharin was employed a main significant effect of conditioning (Paired vs. Yoked) was observed. ^###^ Indicates the significantly higher level of responding of Paired pups when compared to Yoked controls; *p* < 0.0001. When ethanol served as a reinforcer, only Paired pups pre-exposed to PB exhibited significantly higher levels of sensor contacts when compared with the corresponding Yoked controls (^*^); *p* < 0.05. Vertical lines indicate standard errors of the means (SEMs).

Figure 4 depicts the total number of sensor contacts in Paired and Yoked groups reinforced with either intraorally administered saccharin or ethanol. Separate ANOVAs were conducted to analyze learning patterns dependent upon either saccharin (0.1%) or ethanol (3%) reinforcement. In each case, tree-way mixed ANOVAs were used. This analyses were defined by drug treatment as the between factor and conditioning (Paired or Yoked) as well as minutes of evaluation as within factors.

When saccharin was employed as a reinforcer, the ANOVA indicated that conditioning exerted a significant main effect [*F*_(1, 28)_ = 34.82, *p* < 0.0001]. As can be observed in Figure 4, all Paired groups, independently from prior drug experience, showed higher operant responsiveness relative to the corresponding Yoked controls. This result is analogous to those reported when employing saccharin in older infants (Miranda-Morales et al., 2014) or when neonates are reinforced with milk (Arias et al., 2007; Bordner et al., 2008). It is interesting to note that prior drug exposure appears not to affect the learning capability of the organisms nor its overall activity. Relative to the activity rate, Yoked controls pretreated with buffer, ethanol or acetaldehyde showed similar levels of spontaneous sensor contacts.

When ethanol was employed as a reinforcer not all Paired groups differed from the corresponding Yoked controls. The ANOVA revealed a significant main effect of conditioning and a significant interaction between this factor and drug treatment [*F*_(1, 32)_ = 21.75, *p* < 0.0001 and *F*_(2, 32)_ = 3.51, *p* = 0.0419; respectively]. Planned comparisons indicated that only Paired pups treated with a PB control solution had higher sensor contacts that their corresponding Yoked controls. Pretreatment with ethanol or its metabolite appeared to decrease the reinforcement capability of ethanol. Once again, this effect cannot be attributed to motor activity differences across drug pretreatments that can contribute to the probability of sensor contacts. Relative to this issue, all Yoked groups has similar levels of activity.

## Discussion

As stated (see Introduction section) the present study pursued two main goals: (i) the analysis of central ethanol and ACD effects in terms of disruptive effects upon early respiratory plasticity and possible association existing between ethanol and/or ACD central effects leading to thermoregulatory alterations in neonatal rats, and (ii) the analysis of central pre-exposure to the ethanol or its metabolite effect on subsequent seeking behavior of ethanol as a reinforcer in an operant task in neonate rats (Arias et al., 2007; Bordner et al., 2008; March et al., 2009; Miranda-Morales et al., 2014).

Ethanol and ACD doses were chosen according to previous literature indicating analogous effects at least when considering the motivational properties of these drugs (Nizhnikov et al., 2006, 2007; March et al., 2013a,b). When doing so, during PDs 2 and 4, neonates exhibited a respiratory depression when administered with ACD and tested only 5 min after drug administration. This effect was clear at the beginning of the testing procedure (minute 1) relative to control pups administered with PB. Pups treated with ethanol exhibited intermediate values relative to the above mentioned groups. These effects were observed despite the fact that at this post-administration time, respiration rates were very low across groups (see Figure 2). Preliminary pilot studies performed with untreated animals confirmed that the mere handling of the neonates is enough to alter respiratory frequencies when evaluations are temporally close to this manipulation. The values obtained in these untreated pups were found to be almost identical to the PB controls here utilized.

It was also observed that respiratory frequencies increased at 60 and 120 min post-administration time (Figure 1) and neither ethanol nor ACD exerted depressant effects relative to controls. Relative to ACD, these null results may indicate that further pharmacokinetic processes (e.g., ACD conversion into acetate) partially or completely reduce brain concentrations of the metabolite (Quertemont and Didone, 2006; Zimatkin et al., 2006; Hipolito et al., 2007). The fact that ethanol was never found to produce significant respiratory decrements may be related with the dose here employed. Under the present experimental circumstances, it is not possible to determine whether the 100 mg% dose is sufficient to negatively act upon respiratory plasticity or generate, via oxidative processes, ACD levels capable of disrupting breathing patterns. Relative to this dose-related problem, and as previously stated, it is interesting to note that pups tested shortly after receiving brain ethanol administration, exhibited a trend toward a reduction in breathing frequencies relative to controls but not as profound as the group treated with ACD (Figure 2).

At PD5, pups were re-exposed to the testing chamber without receiving any explicit drug treatment. ACD pretreated neonates were found to show heightened respiratory frequencies relative to the remaining groups (Figure 3). We cannot discard the possibility that prior administrations of the metabolite disrupted the respiratory system causing hyperventilation. Nevertheless, a second hypothesis seems plausible. The effect at PD5 (high respiration rates) was opposite relative to the one observed at PDs 2 and 4 (low respiration rates). This apparent contradiction is in agreement with the establishment of learned tolerance to drugs of abuse where conditioned stimuli elicit neurally-mediated homeostatic responses that serve to reduce a specific perturbation (Woods and Ramsay, 2000). Some studies have shown that ambient cues associated with the depressant effects of ethanol appear to modulate the effects of the drug upon respiratory plasticity (Cullere et al., 2015; Macchione et al., 2016; Acevedo et al., 2017). This modulation is related with classical conditioning learning where ambient cues such as the testing environment or a specific odorant (e.g., ethanol odor perceived in the amniotic fluid or as an ambient odor) are associated with the unconditioned effect of the drug. Similar learning processes have been observed in 2-day-old mice when olfactory cues associated with maternal care resulted in heightened conditioned respiratory responses (Durand et al., 2003). When utilizing peripheral ethanol in developing rats, conditioned responses are *isodirectional* relative to the depressant effects upon respiration. In accordance with the systematic review of Eikelboom and Stewart (1982), physiological disruptions mediated by the central nervous system are associated with conditioned stimuli which later elicit *compensatory* conditioned reactivity. If the drug acts on afferent pathways of the brain, the associative process results in isodirectional conditioned responses. In agreement with their analysis and predictions based on specific feedback mechanisms, cues associated with respiratory depressions caused by peripheral (i.g.) ethanol administrations, latter elicit isodirectional learned responses (Macchione et al., 2016). As observed in the present experiment, contextual cues associated with central-nervous-system-mediated respiratory depressions caused by ACD, cause the opposite (probably compensatory) effect.

As previously stated (see Introduction), thermoregulatory disruptions can determine or modulate respiratory depressions. When considering neonatal thermal responsiveness at PDs 2 and 4, it was clear that soon after intracisternal administrations (5 min), body temperatures were low when compared to those recorded at post-administrations time 60 or 120 min (Table 1). Stress-related effects of the administration procedure or even the temperature of the solutions injected into the cisterna magna are factors which can account for this phenomenon. As mentioned, at the earlier post-administration time (5 min) we also observed very low breathing frequencies; a result which argues in favor of the modulatory effects of thermoregulation upon breathing. Yet, drug treatment affected breathing but not thermal responsiveness; a result that argues in favor of early breathing disruptions caused by ACD independently from thermal alterations. At PD5, when tests were performed without any prior administration procedure, temperatures were similar across all groups but as stated, ACD pre-exposed neonates exhibited heightened respiratory rates. Once again, this phenomenon favors the hypothesis that ACD respiratory effects across the experiment were not related with temperature variations. Nevertheless, when taken into account that the administration procedure does affect thermoregulation and that all breathing tests were performed in chambers maintained at 31–32°C, a possible association between thermal and breathing disruptions should not be completely overruled.

The second major goal of the study attempted to elucidate whether prior exposure to central ethanol or ACD impacts upon operant conditioning processes where ethanol or saccharin serve as positive intraoral reinforcers. The results obtained with saccharin confirmed the rapid learning capability of neonates that has been reported when utilizing alternative sweet reinforcer or milk (Arias et al., 2007; Bordner et al., 2008; March et al., 2009). Independently of prior drug condition, pups exposed to the explicit contingency between sensor contacts and saccharin intraoral administration (Paired groups), exhibited relative to Yoked controls, a significantly higher number of responses. This pattern of results was not observed when ethanol served as a reinforcer. Once again, Paired pups pre-exposed to the buffer control solution significantly differed from the corresponding Yoked control group. As in previous studies, neonates without any specific prior drug experience rapidly learn to self-administer an ethanol solution (Bordner et al., 2008; March et al., 2009). This was not the case when neonates were centrally administered with ethanol or ACD (PDs 2 and 4) prior to the assessment of response-stimulus learning associations (PD5). When using these drugs Paired pups did not differ from Yoked controls. Furthermore, Paired pups with a prior history of ACD administrations differed from Paired pups pretreated with the buffer solution. As in the case of respiratory frequencies, Paired subjects pre-exposed to central ethanol, exhibited intermediate levels of responding relative to the two remaining drug-related Paired conditions (ACD or PB).

The results obtained with saccharin indicate that neither ethanol nor ACD pre-exposure altered learning capabilities of the neonates. Therefore, the absence of operant conditioning observed in Paired pups reinforced with ethanol that were previously treated with ethanol or ACD, argues against deleterious effects of these drugs upon the learning process itself. Two hypotheses appear pertinent when addressing the lack of significant learning in Paired subjects pre-exposed to ethanol or ACD and subsequently reinforced with ethanol. Both of them are based on the direct action of ACD in the central nervous system and the biotransformation of ethanol into its principal metabolite via the central catalase system. Favoring the possibility of rapid ethanol metabolism in the neonatal brain is the fact that catalase concentrations in the brain of the newborn rat are approximately eight times higher than in adult animals (Del Maestro and McDonald, 1987). The first hypothesis is related with prior findings concerning altered motor neonatal activity induced by central ACD administration. March et al. (2013a) reported that a single neonatal central administration of ACD (0.52 μmol) exerts a sedative effect upon motor activity. Hence, it is difficult to discard the possibility that sequential administrations of lower ACD doses (0.35 μmol) or of ethanol (100 mg%) into the brain, sensitizes the organism to later sedative of effects of ethanol administered during the operant conditioning task. Nevertheless, this hypothesis is not supported by the following observations. When focusing on the motor activity of Yoked controls (i.e., number of spontaneous sensor contacts) that also received ethanol as a function of the activity of the corresponding Paired neonates, no specific effects of prior drug treatment were detected. The second observation arguing against the motor-related hypothesis is that March et al. (2013a) also found that late prenatal exposure to ethanol generates tolerance rather than sensitization to the depressant of ACD. As stated, this metabolite is likely to be formed due to brain metabolic processes when neonates were exposed to ethanol during the operant task. Yet, when considering both ethanol and ACD pre-exposure effects upon operant performance, we cannot completely discard an alternative possibility of sensitization effects related with anxiogenic or antianxiety effects of both drugs. The arousal state involved in the acquisition of the operant response could be affected by either of these effects. Infants are sensitive to ethanol's antianxiety effects (Pautassi et al., 2007) but there is still no empirical evidence supporting a sensitization effect as a function of prior ethanol treatment during early development. On the contrary, in neonates, exposure to moderate or high ethanol doses seem to potentiate later states of anxiety (Brolese et al., 2014; Baculis et al., 2015). In adults, when considering centrally administered ACD, inhibition of the catalase system or when sequestering this metabolite, the results argue in favor of anxiogenic rather than antianxiety effects (Correa et al., 2008). Taken these considerations into account, sensitization to ethanol's or ACD's anti-anxiety effects does not seem to adequately account for the described disruptions in operant learning processes. The possibility of sensitization to anxiogenic effects of early ethanol or ACD central administration should not be discarded. The second hypothesis is related with the consequences of drug pre-exposure upon subsequent ethanol's motivational properties. Only one conditioning trial has been utilized when central ethanol or ACD are observed to exert positive reinforcing effects in neonates (Nizhnikov et al., 2007; March et al., 2013a,b). So far we ignore if increasing the number of doses results in the recruitment of aversive properties of these psychopharmacological agents or maximizes the possibility of an unconditioned stimulus pre-exposure effect that later competes with the contingency existing between operant responses and ethanol reinforcement. In either case, subsequent ethanol positive reinforcing effects are likely to be devalued. Most importantly, it is necessary to consider that during PDs 2 and 4, these drugs were intracisternally administered. This procedure, the additional handling of the neonate and the isolation from the mother can be viewed as significant aversive stressors (Molina et al., 2000; Hofer et al., 2002; Pautassi et al., 2007). Hence, during these days the effects of the drugs were contingent with aversive events; an association that may compete with subsequent reinforcing effects of ethanol or its metabolite. Notice that whenever pre-exposure to ethanol has resulted in early sensitization to the reinforcing effects of the drug (Nizhnikov et al., 2006; Pautassi et al., 2012), the initial drug experience occurred during late prenatal life via maternal ethanol administration and without any explicit manipulation of the fetus or its natural environment. In support of the present hypothesis, studies have demonstrated that early in ontogeny, the rat is capable of associating different motivational effects of ethanol (positive reinforcing, aversive or anxiolytic) with aversive and appetitive stimuli such as citric acid and sucrose; respectively. As a result of the nature of the associations, the effects of the drug or of the alternative stimuli are reduced or potentiated (Molina et al., 1996; Pautassi et al., 2006; Cullere et al., 2014). The proposed hypothesis may also apply when considering the heightened respiratory rates (PD5) observed in neonates pre-exposed to acetaldehyde. The interoceptive effects of the metabolite (PDs 2 and 4) were experienced in a distinct context (plethysmograph chamber) immediately following intracisternal administration of the drug and while pups were isolated from the mother. At test (PD5), these pups were again placed in the context and as stated, they exhibited a significant increase in breathing frequencies. This physiological reaction, under the framework of the present hypothesis, may represent an anticipatory physiological response linked with prior experiences involving the context, the interoceptive effects of acetaldehyde and different stressors. Obviously, this hypothesis requires further investigation.

Beyond these considerations, the results of this study argue in favor of centrally mediated respiratory and motivational effects of acetaldehyde during a stage in development comparable to the 3rd human gestational trimester. To our knowledge this is the first study indicating a significant role of the metabolite upon early respiratory plasticity. This phenomenon logically requires further investigation particularly when considering the negative effects of ethanol upon the developing respiratory network (Abel, 1981; Dubois et al., 2013). In conjunction with recently conducted studies in rat fetuses and neonates (Cullere et al., 2015; Macchione et al., 2016; Acevedo et al., 2017), the present results confirm that disruptions of the developing respiratory network are attained even when employing moderate levels of exposure to ethanol, or in the present case, to acetaldehyde. Breathing depressions associated with hypoxemia and bradycardia represent a risk factor in terms of hypoxic ischemic effects upon the developing human brain (Pillekamp et al., 2007). As mentioned, fetal alcohol exposure also represents a risk factor for Sudden Infant Death Syndrome (Burd et al., 2004; O'Leary et al., 2013), a phenomenon that has driven scientific attention toward the effects of the drug upon respiratory plasticity. Hence, when considering breathing disruptions involved in hypoxic ischemic consequences upon the brain and the need to better understand factors that can potentially predispose to a devastating pathology such as the Sudden Infant Death Syndrome, the role of acetaldehyde, following ethanol exposure, deserves further scientific attention. Finally, the present study also indicates that centrally administered acetaldehyde impacts upon later ethanol self-administration patterns operationalized through operant conditioning procedures. From a general perspective, these results validate the notions that the metabolite is a neuroactive agent capable of mediating ethanol's motivational properties (Quertemont et al., 2005; Correa et al., 2012) and that it plays a significant role during early ontogeny in terms of structuring ethanol affinity (March et al., 2013a).

## Author contributions

All authors (MA, GD, OH and JM) meet the following criteria: -contributed to the conception or design of the work, -contributed to the analysis and interpretation of data, -participated in the writing and revision of the draft, and -agree to be accountable for all aspects of the work in ensuring that questions related to the accuracy or integrity of any part of the work are appropriately investigated and resolved.

## Funding

This work was supported by grants from Agencia Nacional de Promoción Científica y Tecnológica (FONCyT, PICT 2011-0999 and PICT 2014-1606), SECyT and CONICET PIP 11220110100763 awarded to JM;.

### Conflict of interest statement

The authors declare that the research was conducted in the absence of any commercial or financial relationships that could be construed as a potential conflict of interest.
